# Highest oxygen consumption prediction by forced vital capacity in athletes

**DOI:** 10.1007/s00421-026-06143-7

**Published:** 2026-02-07

**Authors:** Irma Lorenzo-Capellá, Juan José Ramos-Álvarez, Maria Elena Jiménez-Herranz, Nicola Maffulli, Francisco Javier Calderón-Montero, Ghazi Racil, Enzo Iuliano, Gian Mario Migliaccio, Johnny Padulo

**Affiliations:** 1 Faculty of Education and Health, Camilo José Cela University, Madrid, Spain; 2 School of Sports Medicine, Madrid Complutense University, Madrid, Spain; 3 Department of Trauma and Orthopedic Surgery, Faculty of Medicine and Psychology, Sapienza University of Rome, Rome, Italy; 4 Institute of Science and Technology in Medicine, Guy Hilton Research Centre, School of Medicine, Keele University, Stoke-on-Trent, UK; 5 Centre for Sports and Exercise Medicine, Barts and the London School of Medicine and Surgery, Queen Mary University of London, London, UK; 6 Faculty of Physical Activity and Sport Sciences, INEF, Madrid Polytechnic University, Madrid, Spain; 7 Department of Biological Sciences, Higher Institute of Sport and Physical Education of Gafsa, Gafsa, Tunisia; 8 Research Unit (LR 23JS01) “Sport Performance, Health & Society” Higher Institute of Sport and Physical Education of Ksar Said, Tunis, Tunisia; 9 Department of Theoretical and Applied Sciences, eCampus University, Novedrate, Italy; 10 Department of Human Sciences and Promotion of the Quality of Life, San Raffaele Rome Open University, Rome, Italy; 11 Maxima Performa, Athlete Physiology, Psychology and Nutrition Unit, Milan, Italy; 12 Department of Biomedical Sciences for Health, Università Degli Studi Di Milano, Milan, Italy

**Keywords:** Aerobic capacity, Exercise testing, Forced vital capacity, Predictive equations, Athletic performance, Pulmonary function

## Abstract

**Purpose:**

Static spirometry parameters may offer practical alternatives to estimate maximum oxygen consumption (V̇O_2max_) in athletic populations. This study evaluated forced vital capacity (FVC) as a predictor of V̇O_2max_ across different sports, developing prediction equations for field-based assessment.

**Methods:**

Four hundred twenty-two athletes (324 males, 98 females; age 22.9 ± 8.5 years) from cycling (*n* = 123), swimming (*n* = 68), triathlon (*n* = 60), multisport (*n* = 83), and other sports (*n* = 88) performed spirometry and maximal incremental testing. V̇O_2max_ was directly measured using breath-by-breath gas analysis. LASSO regression identified predictors, with Bland–Altman analysis assessing agreement.

**Results:**

FVC and gender emerged as significant predictors (*R*^2^ = 0.690, *P* < 0.001). The equation V̇O_2max_ (L·min^−1^) = (FVC × 0.61) + (Gender × 0.86) yielded SEE = 0.65 L·min^−1^. Including additional variables (Maximum voluntary ventilation, body weight, age) marginally improved prediction (*R*^2^ = 0.712) but reduced practical utility. Coefficient of variation between measured and predicted values was 12.1%. Sport-specific analysis revealed highest predictive accuracy in swimmers (*R*^2^ = 0.893).

**Conclusion:**

FVC provides reasonable population-level V̇O_2max_ estimates in athletes, though individual predictions require caution given substantial unexplained variance (31%). Sport-specific equations, particularly for swimming populations, enhance predictive accuracy. These findings offer practical screening tools for coaches lacking access to metabolic testing equipment, though direct measurement remains the gold standard for individual assessment.

## Introduction

Maximum oxygen consumption (V̇O_2max_) is the gold standard measurement for cardiorespiratory fitness and aerobic performance assessment in both clinical and sports settings (Bassett and Howley 2000; Rabadán et al. 2011; Wagner 2023). In athletic populations, V̇O_2max_ distinguishes aerobic capabilities, particularly in endurance sports where performance directly correlates with oxygen consumption (Martínez-Gómez et al. 2020). However, in intermittent sports such as football or rugby, which tax both the combined aerobic and anaerobic energy systems, V̇O_2max_ remains essential but its relevance more complex, contributing significantly to recovery optimization between high-intensity efforts (Krustrup et al. 2005; Benito et al. 2016; Fu et al. 2021).

Direct V̇O_2max_ measurement typically involves incremental tests to exhaustion with continuous gas exchange monitoring through specialized analyzers (Gao et al. 2021; Magee et al. 2021). While providing high accuracy, this method requires sophisticated laboratory settings, trained personnel, and substantial resources. Additionally, these protocols can be physically demanding, making them potentially unsuitable for certain populations (Hogg et al. 2015). Consequently, numerous indirect methods have gained popularity given their practicality and accessibility.

The physiological determinants of V̇O_2max_ are well established: cardiac output, hemoglobin mass, and peripheral oxygen extraction capacity—including mitochondrial density and muscle capillarization—account for the majority of inter-individual variance in aerobic capacity (Bassett and Howley 2000; Wagner 2023). In healthy trained individuals, the respiratory system is generally not considered a primary limiting factor, as ventilatory capacity typically exceeds metabolic demand even at maximal exercise intensities (Dempsey et al. 2006; Amann 2012). Nevertheless, pulmonary function parameters may still hold predictive value for V̇O_2max_ through indirect mechanisms.

Static spirometric indices such as Forced Vital Capacity (FVC) scale proportionally with body size, sex, and overall physiological development, reflecting individual variations in total lung volume and thoracic dimensions (Dempsey et al. 2006; Amann 2012). Because absolute V̇O_2max_ (L·min⁻^1^) is itself strongly influenced by body mass, lean mass, and sex-related differences in oxygen transport capacity, FVC may serve as an integrative anthropometric proxy capturing these shared determinants. This does not imply a direct mechanistic link between static lung volumes and aerobic metabolism but rather suggests that FVC could function as a practical surrogate variable for field-based V̇O_2max_ estimation when direct measurement is unfeasible (Hackett 2020; Lorenzo-Capellá et al. 2025b). Additionally, maximum Voluntary Ventilation (MVV), representing the maximal volume of air an individual can voluntarily move in one minute, serves as an indirect assessment of respiratory muscle strength and endurance (Neder et al. 1999). Unlike static volumes, MVV reflects dynamic ventilatory function and may capture training-induced adaptations in respiratory muscle performance.

Athletes across different sports demonstrate unique adaptations in pulmonary function (Durmic et al. 2015; Mazic et al. 2015). Exercise training enhances respiratory muscle endurance and strength, reduces bronchial resistance, and promotes increased lung elasticity and alveolar expansion (Özdal 2016; Hackett 2020). Previous research has established predictive relationships between FEV_1_ and MVV in athletes, with sport-specific equations providing greater accuracy (Sikora et al. 2024; Lorenzo-Capellá et al. 2025a). However, the specific predictive value of FVC for V̇O_2max_ across various athletic populations remains insufficiently explored. Therefore, this study aimed to evaluate the potential of FVC as a predictor of V̇O_2max_ in athletes from various sports, recognizing that any predictive relationship likely reflects shared anthropometric and sex-related variance rather than direct respiratory limitation of aerobic capacity.

## Methods

### Participants

This retrospective, quasi-experimental study analyzed data from 422 athletes selected from 2,123 participants who attended our center between 2012 and 2019 years. Inclusion criteria were: (a) correct performance of both spirometry and cardiovascular stress tests, and (b) regular sports participation (minimum 6 h/week for 3 + years). Sample characteristics are presented in Table 1. All protocols followed the Declaration of Helsinki, with written informed consent obtained from all participants.

**Table 1:** Characteristics of the sample

Sample size (n)	422
Age (years)	22.9 ± 8.5 (min–max, 8–56)
Body mass (kg)	66.9 ± 12.2 (min–max, 28–96)
Stature (cm)	173.3 ± 11.0 (min–max, 129–206)
Gender (n)	Females (98) and Males (324)
Sport practiced (n)	Cycling athletes (123) Swimming athletes (68) Triathlon athletes (60) Athletes practicing multiple sports (83) Athletes practicing other sports (no multiple sports)* (88)

Values or Age, Body mass, Stature are expressed as means ± Standard deviation and range in brackets; Values of Sample size, Gender and Sport practiced are expressed as numerosity in brackets; * This group included athletes who practiced a single sport, but the total number of athletes practicing that specific sport was < 23. Therefore, due to the low sample size, these athletes were considered a single group regardless of the practiced sport

### Spirometry testing

Forced vital capacity (FVC) and forced expiratory volume in one second (FEV_1_) were measured following the American Thoracic Society/European Respiratory Society (ATS/ERS) guidelines (Graham et al. 2019). Participants wore nose clips and performed maximal inspiration followed by forced expiration using a calibrated spirometer (Quark SPIRO, Cosmed SRL, Rome, Italy). The best of two acceptable maneuvers was recorded. Maximum voluntary ventilation (MVV) was assessed using a 12-s protocol with participants encouraged to maintain maximal breathing frequency and depth while viewing real-time visual feedback. Three maneuvers with < 10% variation were obtained, with all MVV values exceeding FEV_1_-estimated values.

### Maximal oxygen consumption testing

V̇O_2max_ was measured using sport-specific ergometers (cycle ergometer for cyclists, treadmill for all others) with continuous incremental protocols until volitional exhaustion. Test termination criteria included: (a) oxygen consumption plateau (< 150 mL·min^−1^ or 2.1 mL·kg·min^−1^ increase); (b) respiratory quotient ≥ 1.10; (c) achieving age-predicted maximal heart rate; or (d) volitional exhaustion. Gas exchange was measured breath-by-breath using an automated oxygen analyzer (Jaeger Oxycon Pro, Viasys Healthcare, Germany) with bidirectional digital volume transducer (Triple V^®^). The oxygen analyzer measured gas proportion using the differential paramagnetic principle, while carbon dioxide was analyzed via infrared absorption. Heart rate was continuously recorded via 12-lead ECG (Viasys Healthcare, Germany). All tests were performed under standardized environmental conditions (BTPS), with *participants abstaining from intense exercise and stimulants for 24 h prior.*

### Statistical analysis

Data normality was assessed using Shapiro–Wilk tests. Least Absolute Shrinkage and Selection Operator (LASSO) Regression were used to identify significant predictors of absolute V̇O_2max_ (L·min^−1^) from FEV_1_, FVC, MVV, age, gender, body mass index (BMI), and body weight. The coefficients obtained from the regression analyses were used to compute the equation to estimate V̇O_2max_. Variance inflation factor (VIF) assessed multicollinearity. The Lambda value (λ) for LASSO regression was determined using Cross-Validation method and the λ value that minimizes prediction error was used. Subsequently, Automatic Linear Modeling Technique was used to estimate the unique relative importance of each predictor. Bland–Altman plots evaluated agreement between measured and predicted values. Also, repeated measures ANOVA was performed to compare measured and estimated V̇O_2max_ to verify whether significant differences existed between these values. Finally, the coefficient of variation between measured and estimated V̇O_2max_ was calculated. Sport-specific analyses were performed for groups with adequate sample sizes. Significance was set at *P* < 0.05. Analyses were performed using SPSS v.26 (IBM Corp., Armonk, NY, USA) and R software v.4.5.2 (R Core Team, Vienna, Austria).

## Results

### Participant characteristics

Table 2 presents participant demographics. The swimming cohort was significantly younger (age 13.4 ± 3.5 years) than other sports (*P* < 0.001) and showed a significantly different gender distribution (*P* < 0.001).

**Table 2:** Participant characteristics and cardiorespiratory parameters by sport discipline

Sample size (n)	422
*Gender (n)*	Females (98) and Males (324)
Cycling athletes	Females (14) and Males (109)
Swimming athletes	Females (43) and Males (25)*
Triathlon athletes	Females (10) and Males (50)
Athletes practicing multiple sports	Females (12) and Males (71)
Athletes practicing other sports	Females (19) and Males (69)
*Age (years)*	22.90 ± 8.50 (min–max, 8.00–56.00); −1.75 to 3.89
Males	24.24 ± 8.44 (min–max, 9.00–56.00); −1.81 to 3.76
Females	18.32 ± 6.87 (min–max, 8.00–47.00); −1.50 to 4.17
Cycling athletes	23.40 ± 7.78 (min–max, 14.00–52.00); −1.21 to 3.68
Swimming athletes	13.44 ± 3.47 (min–max, 8.00–26.00); −1.57 to 3.62#
Triathlon athletes	28.48 ± 6.94 (min–max, 17.00–55.00); −1.65 to 3.82
Athletes practicing multiple sports	25.80 ± 8.48 (min–max,18.00–56.00); −0.92 to 3.56
Athletes practicing other sports	22.82 ± 7.31 (min–max, 12.00–56.00); −1.48 to 4.54
*V̇O*_*2max*_* measured (L·min*^*−1*^*)*	3.86 ± 1.16 (min–max, 0.76–6.29); −2.67 to 2.09
Males	4.30 ± 0.85 (min–max,1.48–6.29); −3.32 to 2.34
Females	2.40 ± 0.79 (min–max, 0.76–4.49); −2.08 to 2.65
Cycling athletes	4.62 ± 0.85 (min–max, 2.48–6.29); −2.52 to 1.96
Swimming athletes	2.32 ± 0.99 (min–max, 0.76–4.78); −1.58 to 2.48
Triathlon athletes	4.19 ± 0.81 (min–max, 2.53–6.22); −2.05 to 2.51
Athletes practicing multiple sports	3.65 ± 0.87 (min–max, 1.53–5.54); −2.44 to 2.17
Athletes practicing other sports	3.96 ± 0.88 (min–max, 1.85–5.67); −2.4 to 1.94
*FVC (liters)*	5.04 ± 1.13 (min–max, 1.61–8.17); −3.04 to 2.77
Males	5.44 ± 0.9 (min–max, 2.31–8.17); −3.48 to 3.03
Females	3.72 ± 0.79 (min–max, 1.61–5.3); −2.67 to 2.00
Cycling athletes	5.27 ± 0.86 (min–max, 3.06–7.34); −2.57 to 2.41
Swimming athletes	3.72 ± 1.18 (min–max, 1.61–6.68); −1.79 to 2.51
Triathlon athletes	5.40 ± 0.97 (min–max, 3.55–7.93); −1.91 to 2.61
Athletes practicing multiple sports	5.21 ± 0.97 (min–max, 2.79–7.27); −2.49 to 2.12
Athletes practicing other sports	5.33 ± 0.98 (min–max, 3.27–8.17); −2.1 to 2.9
*FEV*_*1*_* (liters)*	4.29 ± 1.00 (min–max, 1.37–6.86); −2.92 to 2.57
Males	4.62 ± 0.83 (min–max, 1.46–6.86); −3.81 to 2.70
Females	3.22 ± 0.72 (min–max, 1.37–4.67); −2.57 to 2.01
Cycling athletes	4.50 ± 0.84 (min–max, 1.75–6.86); −3.27 to 2.81
Swimming athletes	3.26 ± 1.09 (min–max, 1.37–5.95); −1.73 to 2.47
Triathlon athletes	4.54 ± 0.89 (min–max, 2.42–6.47); −2.38 to 2.17
Athletes practicing multiple sports	4.45 ± 0.85 (min–max,1.53–5.99); −3.44 to 1.81
Athletes practicing other sports	4.49 ± 0.83 (min–max,2.67–6.47); −2.19 to 2.39
*MVV (liters)*	156.30 ± 43.24 (min–max, 45.38–258.98); −2.57 to 2.37
Males	171.07 ± 35.21 (min–max, 48.50–258.98); −3.48 to 2.50
Females	107.49 ± 29.17 (min–max, 45.38–159.54); −2.13 to 1.78
Cycling athletes	165.01 ± 37.75 (min–max, 48.50–258.98); −3.09 to 2.49
Swimming athletes	105.38 ± 39.74 (min–max, 45.38–217.41); −1.51 to 2.82
Triathlon athletes	173.9 ± 31.13 (min–max, 99.10–235.74); −2.4 to 1.99
Athletes practicing multiple sports	168.7 ± 35.78 (min–max, 78.96–244.93); −2.51 to 2.13
Athletes practicing other sports	159.8 ± 38.26 (min–max, 76.35–255.54); −2.18 to 2.50
*Body mass (kilogram)*	66.95 ± 12.21 (min–max, 27.80–96.20); −3.21 to 2.4
Males	70.66 ± 9.82 (min–max, 31.10–96.20); −4.03 to 2.6
Females	54.68 ± 11.30 (min–max, 27.80–89.00); −2.38 to 3.04
Cycling athletes	68.13 ± 8.18 (min–max, 49.70–93.40); −2.25 to 3.09
Swimming athletes	52.33 ± 13.82 (min–max, 27.80–86.30); −1.77 to 2.46
Triathlon athletes	68.65 ± 9.09 (min–max, 51.20–88.00); −1.92 to 2.13
Athletes practicing multiple sports	71.56 ± 10.89 (min–max, 47.00–93.00); −2.26 to 1.97
Athletes practicing other sports	71.09 ± 10.19 (min–max, 41.00–96.20); −2.95 to 2.46
*BMI (body mass/stature*^*−2*^*)*	22.10 ± 2.38 (min–max,14.94–28.39); −3.01 to 2.64
Males	22.55 ± 2.23 (min–max,14.94–28.39); −3.41 to 2.62
Females	20.58 ± 2.23 (min–max, 15.90–26.20); −2.10 to 2.52
Cycling athletes	22.3 ± 2.13 (min–max, 17.17–28.17); −2.41 to 2.76
Swimming athletes	19.89 ± 2.44 (min–max,14.94–26.17); −2.03 to 2.57
Triathlon athletes	22.25 ± 1.71 (min–max, 18.81–26.55); −2.01 to 2.51
Athletes practicing multiple sports	23.11 ± 2.48 (min–max, 16.96–28.39); −2.48 to 2.13
Athletes practicing other sports	22.44 ± 1.94 (min–max, 17.31–26.39); −2.64 to 2.04

Values of Gender are expressed as numerosity in brackets. All the other values (Age, V̇O_2max_ measured, FVC, etc.) are expressed as means ± Standard deviation, range (in brackets) and Z-score of the range (after the semicolon); * Swimmers group significantly differs from other sport groups for gender ratio; ^#^ Swimmers group was significantly younger than other sports groups

### $\dot{V}$O_2max_ prediction from spirometry data

Absolute V̇O_2max_ (L·min^−1^) showed superior predictability compared to relative values (mL·kg·min^−1^). Shapiro–Wilk test confirmed normal distribution of residuals (W = 0.996, *P* = 0.086). The Cross-Validation analysis indicated the optimal λ value to minimize prediction error was = 0.0032 and a LASSO regression was performed with this λ value. LASSO regression identified FVC, gender, MVV, body weight, and age as significant predictors (all with p < 0.05), while BMI and FEV₁ were excluded. Consequently, using the coefficients from the analyses, the following full model equation was obtained:

$$\begin{aligned} \dot{V}O_{2} \max {\mkern 1mu} \left( {L \cdot \min ^{{ - 1}} } \right){\mkern 1mu} & = {\mkern 1mu} \left( {FVC{\mkern 1mu} \times {\mkern 1mu} 0.38} \right){\mkern 1mu} + {\mkern 1mu} \left( {Gender{\mkern 1mu} \times {\mkern 1mu} 0.75} \right){\mkern 1mu} + {\mkern 1mu} \left( {MVV{\mkern 1mu} \times {\mkern 1mu} 0.005} \right){\mkern 1mu} \\ & + {\mkern 1mu} \left( {Body{\mkern 1mu} Weight{\mkern 1mu} \times {\mkern 1mu} 0.017} \right){\mkern 1mu} - {\mkern 1mu} \left( {Age{\mkern 1mu} \times {\mkern 1mu} 0.014} \right){\mkern 1mu} - {\mkern 1mu} 0.22 \\ \end{aligned}$$

with R^2^ = 0.715, SEE = 0.62 L·min^−1^, and negligible multicollinearity (VIF = 1.3–3.8). The Automatic Linear Modeling Technique indicated the following relative importance of each predictor (metrics are normalized to sum to 100%): FVC = 39%, Gender = 37%, MVV = 10%, Body Weight = 10%, and Age = 4%.

On the basis of these results, a simplified model using only FVC and gender was also calculated, achieving almost comparable prediction capability (R^2^ = 0.690, SEE = 0.65 L·min^−1^):

$$\dot{V}O_{2} \max \, \left( {L\cdot\min^{ - 1} } \right) \, = \, \left( {FVC \, \times \, 0.61} \right) \, + \, \left( {Gender \, \times \, 0.85} \right) \, + \, 0.16$$

Where FVC is in liters and gender is coded 1 for males, 0 for females. This simplifies to:

$$Males \, \dot{V}O_{2} \max \, \left( {L\cdot\min^{ - 1} } \right) \, = \, \left( {FVC \, \times \, 0.61} \right) \, + \, 1.01$$

$$Females \, \dot{V}O_{2} \max \, \left( {L\cdot\min^{ - 1} } \right) \, = \, \left( {FVC \, \times \, 0.61} \right) \, + \, 0.16$$

In this case, the Cross-Validation analysis indicated, for the LASSO regression, an optimal λ value = 0.0061 to minimize prediction error.

### Agreement analysis

**Fig. 1:**
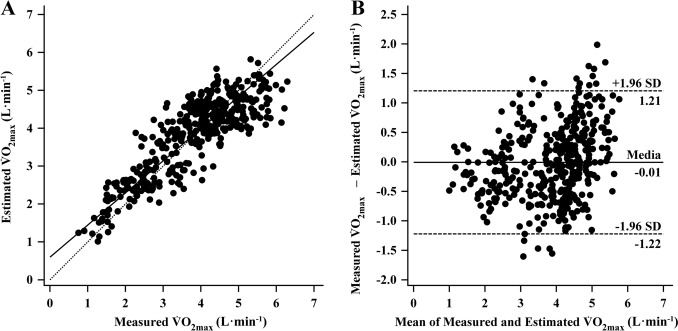
Correlation and Bland–Altman plots for V̇O_2max_ prediction using full model equation V̇O_2max_ (L·min^−1^) = (FVC × 0.38) + (Gender × 0.75) + (MVV × 0.005) + (Body Weight × 0.017)−(Age × 0.014)−0.24: **A** Correlation between measured and estimated V̇O_2max_. Bold line represents correlation trend, while dotted line represents equivalence (y = x); **B** Bland–Altman plot showing mean of measurements (x-axis) versus difference between measurements (y-axis). Bold line indicates mean bias, while dashed lines indicate 1.96 SD limits of agreement

When considering the equation including the full model, the correlation between measured and predicted V̇O_2max_ was r = 0.846 (*P* < 0.001; Fig. 1—Panel A), with coefficient of variation 12.1%. Bland–Altman analysis revealed mean bias of −0.01 L·min^−1^ with 1.96 SD limits ranging from –1.22 to + 1.21 L·min^−1^ (Fig. 1—Panel B). Repeated measures ANOVA showed no significant differences between measured V̇O_2max_ and the V̇O_2max_ estimated with the full model equation (F_(1,421)_ = 0.086; *P* = 0.769).

**Fig. 2:**
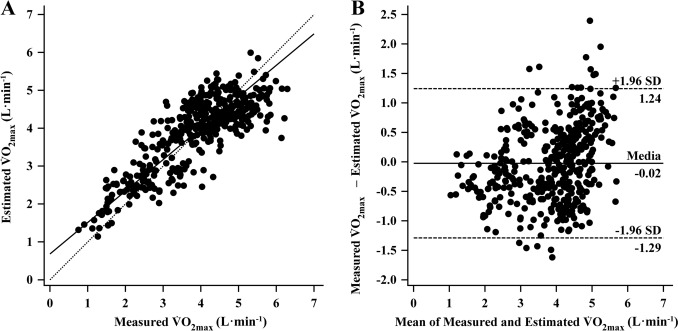
Correlation and Bland–Altman plots for V̇O_2max_ prediction using the simplified equation V̇O_2max_ (L·min^−1^) = (FVC × 0.61) + (Gender × 0.86) + 0.13: **A** Correlation between measured and estimated V̇O_2max_. Bold line represents correlation trend, while dotted line represents equivalence (y = x); **B** Bland–Altman plot showing mean of measurements (x-axis) versus difference between measurements (y-axis). Bold line indicates mean bias, while dashed lines indicate 1.96 SD limits of agreement

When considering the short equation including FVC and gender only, the correlation between measured and predicted V̇O_2max_ was r = 0.831 (*P* < 0.001; Fig. 2—Panel A), with a coefficient of variation of 12.9%. Bland–Altman analysis revealed mean bias of −0.02 L·min^−1^ with 1.96 SD limits ranging from –1.29 to + 1.24 L·min^−1^ (Fig. 2—Panel B). Repeated measures ANOVA showed no significant differences between measured V̇O_2max_ and the V̇O_2max_ estimated with the short equation (F_(1,421)_ = 0.620; *P* = 0.432).

### Sport-specific analyses

When considering Sport-specific regression LASSO regression analyses showed variable predictive capacity:

$$\begin{aligned} Swimming:\dot{V}O_{2} \max \left( {L \cdot \min ^{{ - 1}} } \right) & = \left( {FVC{\mkern 1mu} \times 0.23} \right) + \left( {Gender{\mkern 1mu} \times {\mkern 1mu} 0.49} \right) \\ & + \left( {MVV{\mkern 1mu} \times {\mkern 1mu} 0.009} \right) + \left( {Body\,{\mkern 1mu} Weight{\mkern 1mu} \times {\mkern 1mu} 0.014} \right) - 0.35;{\mkern 1mu} \\ & R^{2} = 0.899,SEE = 0.31{\mkern 1mu} L \cdot \min ^{{ - 1}} \\ \end{aligned}$$

$$\begin{aligned} Cycling:{\mkern 1mu} \dot{V}O_{2} \max \left( {L \cdot \min ^{{ - 1}} } \right) & = {\mkern 1mu} \left( {FVC{\mkern 1mu} \times {\mkern 1mu} 0.39} \right) + \left( {Gender \times 0.54} \right) + \left( {MVV{\mkern 1mu} \times {\mkern 1mu} 0.004} \right) \\ & + \left( {Body\,Weight\,{\mkern 1mu} \times {\mkern 1mu} 0.024} \right) - \left( {Age \times {\mkern 1mu} 0.031} \right) + 0.55;{\mkern 1mu} \\ & R^{2} = 0.675,SEE = 0.49L \cdot \min ^{{ - 1}} \\ \end{aligned}$$

$$\begin{aligned} Triathlon:\dot{V}O_{2} \max \left( {L \cdot \min ^{{ - 1}} } \right) & = \left( {FVC \times 0.21} \right) + \left( {Gender \times 0.51} \right) + \left( {MVV \times 0.006} \right) \\ & + \left( {Body\,{\mkern 1mu} Weight \times 0.015} \right) - \left( {Age \times 0.003} \right) + 0.63; \\ & R^{2} = 0.632,SEE = 0.50L \cdot \min ^{{ - 1}} \\ \end{aligned}$$

$$\begin{aligned} Multisport:\dot{V}O_{2} \max \left( {L \cdot \min ^{{ - 1}} } \right) & = \left( {FVC \times 0.25} \right) + \left( {Gender \times 0.78} \right) \\ & + \left( {Body\,Weight \times 0.025} \right) - \left( {Age \times 0.025} \right) + 0.61; \\ & R^{2} = 0.704,SEE = 0.48L \cdot \min ^{{ - 1}} \\ \end{aligned}$$

## Discussion

This investigation provides insights into the predictive relationship between pulmonary function parameters and aerobic capacity in athletic populations. FVC demonstrated a moderate association with V̇O_2max_ (R^2^ = 0.690), indicating that approximately 31% of variance remains unexplained. The simplified equation may offer limited practical utility for population-level screening in field settings, though substantial prediction uncertainty precludes reliable individual assessment. However, several mechanistic and methodological considerations warrant careful examination.

###  Physiological basis of the FVC-$\dot{V}$O_2max_ relationship 

The observed association between FVC and V̇O_2max_ should not be interpreted as evidence of direct mechanistic coupling between static lung volumes and aerobic metabolism. The primary physiological determinants of V̇O_2max_ cardiac output, hemoglobin mass, mitochondrial oxidative capacity, and muscle capillarization—are structurally unrelated to static spirometric indices (Bassett and Howley 2000; Dempsey et al. 2006; Amann 2012; Wagner 2023). In healthy trained individuals, ventilatory capacity typically exceeds metabolic demand even at maximal exercise intensities, and the respiratory system is generally not considered a primary limiting factor for aerobic performance.

The predictive value of FVC likely reflects its role as an integrative anthropometric proxy. FVC scales proportionally with body size, thoracic dimensions, and sex-related differences—variables that also strongly influence absolute V̇O_2max_ (L·min⁻^1^). The Automatic Linear Modeling analysis revealed that FVC (39%) and gender (37%) together accounted for 76% of the relative predictor importance, supporting the interpretation that FVC captures shared variance with anthropometric and sex-related determinants rather than independent respiratory contributions to aerobic capacity.

The substantial unexplained variance (31%) corresponds to the non-respiratory determinants of V̇O_2max_ that static spirometry cannot capture. Individual differences in stroke volume, blood oxygen-carrying capacity, peripheral oxygen extraction, and oxidative enzyme activity represent the mechanistic factors underlying aerobic performance that lie outside the predictive scope of pulmonary function testing. This finding corroborates recent work demonstrating that dynamic respiratory mechanics during exercise provide additional predictive value beyond static spirometry (Milne et al. 2020).

The role of the respiratory system as a potential limiting factor in elite athletes has been described particularly through the lens of exercise-induced respiratory muscle fatigue and consequent metaboreflex activation (Dempsey et al. 2006; Amann 2012). The exclusion of FEV_1_ from our predictive model, despite its established relationship with MVV, suggests that rate-limiting aspects of forced expiration may be less relevant to steady-state aerobic metabolism than total ventilatory capacity. However, this finding should be interpreted cautiously, as it does not imply that FVC directly determines aerobic capacity, but rather that FVC may better capture the anthropometric scaling factors shared *with V̇O*_*2max*_* (*Romer and Polkey 2008; Dominelli and Sheel 2019*).*

### Sport-specific adaptations

The substantial variation in predictive accuracy across sports may reflect sport-specific respiratory adaptations that our current FVC-based model does not fully capture. The swimming cohort showed higher predictive accuracy (R^2^ = 0.899), although this finding warrants cautious interpretation. This subgroup was characterized by younger age (13.4 ± 3.5 years), smaller sample size (*n* = 68), and unbalanced sex distribution (63% female). The elevated R^2^ may substantially reflect developmental covariance between lung growth and aerobic capacity during adolescence rather than sport-specific respiratory adaptations. During puberty, both FVC and absolute V̇O_2max_ increase in parallel with somatic growth, potentially inflating the correlation independently of any training-induced mechanism. Consequently, the swimming-specific equation should be considered preliminary and requires validation in adult, sex-balanced cohorts before practical application. The aquatic training environment does impose distinctive respiratory constraints through hydrostatic pressure and enforced breathing patterns (Lomax and McConnell 2003), but disentangling these sport-specific effects from maturational confounding is not possible with the present cross-sectional data.

Cyclists demonstrated moderate predictive capacity (R^2^ = 0.675), possibly reflecting the influenced seated posture on respiratory mechanics and the emphasis of the sport on sustained aerobic power rather than maximal ventilatory capacity (Millet et al. 2009). The failure to develop robust sport-specific equations for all disciplines represents a limitation that future investigations should address through larger, sport-stratified samples.

### Clinical and practical applications

The equations developed herein may provide gross estimates of V̇O_2max_ for practitioners lacking access to metabolic testing equipment. However, the prediction uncertainty (SEE = 0.65 L·min⁻^1^, CV = 12%) substantially limits their utility beyond population-level screening. Practitioners must recognize these tools as screening instruments rather than diagnostic replacements. The standard error of estimate (0.65 L·min⁻^1^) translates to 95% prediction intervals of approximately ± 1.27 L·min⁻^1^, representing substantial uncertainty that may exceed ± 25–30% for athletes with lower absolute V̇O_2max_ values. For an athlete with measured V̇O_2max_ of 4.0 L·min⁻^1^, the predicted value could range from approximately 2.7 to 5.3 L·min⁻^1^. This magnitude of error renders the equation unsuitable for detecting training-induced changes, which typically range from 5–15% in already trained individuals. Consequently, direct V̇O_2max_ measurement remains the gold standard when precise individual assessment or longitudinal monitoring is required. The practical utility of these equations is therefore restricted to contexts where: (a) metabolic testing is entirely unavailable, (b) only rough population stratification is needed, or (c) initial screening to identify athletes warranting comprehensive assessment is the primary goal.

### Limitations

Several methodological factors constrain our interpretations. The cross-sectional design precludes causal inferences about training-induced adaptations. The heterogeneous sample, while enhancing external validity, introduces variance that sport-specific models might reduce. The retrospective nature of the dataset (2012–2019) and the pooling of multiple sports with dramatically different age distributions represent important constraints. In particular, the swimming cohort’s age differential (13.4 ± 3.5 years) confounds sport-specific interpretations, as developmental factors influence respiratory mechanics independently of training (Lazovic et al. 2015). Age-related differences in lung size profoundly affect static spirometric indices, and the parallel developmental trajectories of pulmonary and cardiovascular systems during adolescence may artificially inflate predictive correlations in younger cohorts. A stratified reanalysis by maturational status was not feasible given the available data but would strengthen future investigations.

Another important limitation concerns the substantial shared variance between FVC and anthropometric variables. The correlation between FVC and body weight in our sample, combined with the strong predictive contribution of gender, suggests that FVC’s predictive capacity may largely overlap with information already captured by sex and body size. The practical implication is that FVC may function primarily as a convenient integrative index of these variables rather than providing independent respiratory information. Finally, another limitation of the study concerns the possible collinearity of some predictors used in the equation developed in the present study. Despite the low VIF values obtained from the analysis, which indicate a statistically negligible collinearity, it must be noted that FVC is in any case a parameter that may depend on the gender (which resulted in an important predictor along with FVC), age, and weight of a subject. Therefore, some of the results obtained in our equation could present a bias due to this aspect, as there might be an overlap of the predictive effects on V̇O_2max_.

## Conclusions

This investigation identifies FVC as a statistically significant but mechanistically indirect predictor of V̇O_2max_ in athletic populations, offering the simplified equation V̇O_2max_ (L·min^−1^) = (FVC × 0.61) + (Gender × 0.86) for field-based screening when direct measurement proves unfeasible. The predictive relationship likely reflects shared anthropometric and sex-related variance rather than direct respiratory determinants of aerobic capacity. While providing rough population-level estimates, individual predictions require cautious interpretation, given substantial unexplained variance and prediction intervals that may exceed clinically meaningful thresholds.

Sport-specific equations, particularly for swimming, demonstrated higher R^2^ values but should be interpreted with caution given small sample sizes, developmental confounding, and sex imbalances that limit generalizability.

Future research should prioritize: (a) prospective validation in independent cohorts, (b) development of sport-specific equations with adequate sample sizes and balanced sex distributions, (c) stratified analysis by maturational status to disentangle developmental from training effects, (d) quantification of FVC’s unique predictive contribution controlling for anthropometric variables, and (e) integration of dynamic respiratory parameters to enhance predictive accuracy. These prediction equations represent a preliminary contribution toward accessible athlete screening, though direct V̇O_2max_ measurement remains the reference standard for individual assessment, and the limitations of spirometry-based prediction necessitate continued refinement through rigorous scientific inquiry.

## Data Availability

The data are available upon specific and reasonable request through direct contact with the corresponding author.

## Abbreviations

ATS: American thoracic society
ANOVA: Analysis of variance
BMI: Body mass index
ERS: European respiratory society
FVC: Forced vital capacity
FEV_1_: Forced expiratory volume in one second
MVV: Maximum voluntary ventilation
SEE: Standard error of the estimate
VIF: Variance inflation factor
V̇O_2max_: Maximum oxygen consumption
